# SER-109: An Oral Investigational Microbiome Therapeutic for Patients with Recurrent *Clostridioides difficile* Infection (rCDI)

**DOI:** 10.3390/antibiotics11091234

**Published:** 2022-09-10

**Authors:** Sahil Khanna, Matthew Sims, Thomas J. Louie, Monika Fischer, Kerry LaPlante, Jessica Allegretti, Brooke R. Hasson, Allyson T. Fonte, Christopher McChalicher, David S. Ege, Jessica A. Bryant, Timothy J. Straub, Christopher B. Ford, Matthew R. Henn, Elaine E. L. Wang, Lisa von Moltke, Mark H. Wilcox

**Affiliations:** 1 Division of Gastroenterology and Hepatology, Mayo Clinic, Rochester, MN 55905, USA; 2 Section of Infectious Diseases and International Medicine, Department of Internal Medicine, Beaumont, Royal Oak, MI 48073, USA; 3 Department of Internal Medicine and Foundational Medical Studies, Oakland University William Beaumont School of Medicine, Rochester, MI 48309, USA; 4 Cumming School of Medicine, University of Calgary, Calgary, AB T2N 1N4, Canada; 5 Division of Gastroenterology and Hepatology, Indiana University, Indianapolis, IN 46202, USA; 6 Department of Pharmacy Practice, University of Rhode Island, Kingston, RI 02881, USA; 7 Division of Infectious Diseases, Warren Alpert Medical School of Brown University, Providence, RI 02903, USA; 8 Division of Gastroenterology, Brigham and Women’s Hospital, Boston, MA 02115, USA; 9 Seres Therapeutics, Cambridge, MA 02139, USA; 10 University of Leeds, Leeds Teaching Hospitals NHS Trust, Leeds LS1 3EX, UK

**Keywords:** *Clostridioides* *difficile* infection (CDI), SER-109, microbiome therapeutics, Firmicutes, microbial diversity, recurrent CDI

## Abstract

*Clostridioides* *difficile* infection (CDI) is classified as an urgent health threat by the Centers for Disease Control and Prevention (CDC), and affects nearly 500,000 Americans annually. Approximately 20–25% of patients with a primary infection experience a recurrence, and the risk of recurrence increases with subsequent episodes to greater than 40%. The leading risk factor for CDI is broad-spectrum antibiotics, which leads to a loss of microbial diversity and impaired colonization resistance. Current FDA-approved CDI treatment strategies target toxin or toxin-producing bacteria, but do not address microbiome disruption, which is key to the pathogenesis of recurrent CDI. Fecal microbiota transplantation (FMT) reduces the risk of recurrent CDI through the restoration of microbial diversity. However, FDA safety alerts describing hospitalizations and deaths related to pathogen transmission have raised safety concerns with the use of unregulated and unstandardized donor-derived products. SER-109 is an investigational oral microbiome therapeutic composed of purified spore-forming Firmicutes. SER-109 was superior to a placebo in reducing CDI recurrence at Week 8 (12% vs. 40%, respectively; *p* < 0.001) in adults with a history of recurrent CDI with a favorable observed safety profile. Here, we discuss the role of the microbiome in CDI pathogenesis and the clinical development of SER-109, including its rigorous manufacturing process, which mitigates the risk of pathogen transmission. Additionally, we discuss compositional and functional changes in the gastrointestinal microbiome in patients with recurrent CDI following treatment with SER-109 that are critical to a sustained clinical response.

## 1. Introduction

*Clostridioides difficile* (*C. difficile*) is the leading cause of healthcare-associated infections in the US [1,2], and was classified as one of the greatest microbial threats to human health by the Centers for Disease Control and Prevention (CDC) in 2013 and 2019. Clinical manifestations of *C. difficile* infection (CDI) range from mild diarrhea to life-threatening colitis. The all-cause mortality rate is estimated to be 11.8–38% [3,4] with 20,500 associated deaths in 2017 [5]. The economic burden of CDI is estimated to be up to USD 5.4 billion annually in the US and primarily driven by hospitalization costs [6].

### 1.1. Role of the Microbiome in Recurrent CDI

**Figure 1:**
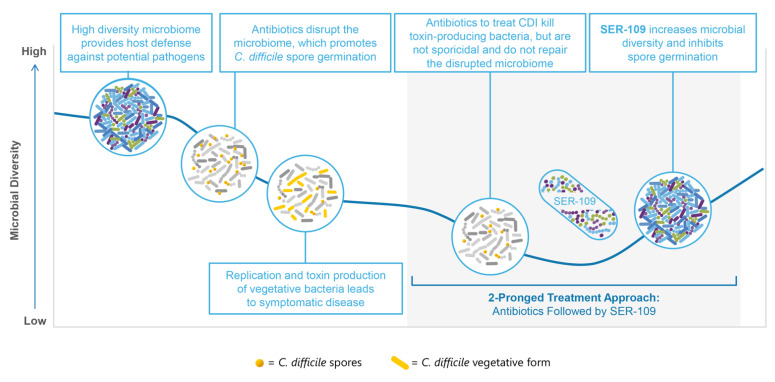
*C. difficile* infection (CDI) is a two-hit process requiring a 2-pronged treatment approach.

The pathogenesis of CDI typically occurs as a two-hit process: (1) the disruption of the microbiome, a diverse ecosystem that provides essential functions for the host; and (2) exposure to *C. difficile* spores (Figure 1) [7,8]. The primary risk factor for disease development is antibiotic use, which contributes to the pathophysiology of CDI by creating ecologic gaps within the microbiome. The loss of microbial diversity reduces colonization resistance and negatively impacts microbe-associated functions that are key to host defense [9,10]. Other risk factors for CDI and/or recurrent CDI (rCDI) include age ≥ 65, female sex, concomitant comorbidities, exposure to antibiotics after treatment for CDI, and proton pump inhibitor use [11,12,13,14]. A leading risk factor for recurrent CDI is a history of recurrence with recurrence rates of up to 49% after completion of CDI-targeted antibiotics [15].

The gastrointestinal microbiome plays an important role in human health, including resistance to colonization by pathogens such as *C. difficile* [9]. In a disrupted microbiome, there is an increase in the abundance of proinflammatory Gram-negative Proteobacteria and a decline in the abundance of beneficial spore-forming Firmicutes species that play a dominant role in gastrointestinal health [9,10]. The loss of Gram-positive Firmicutes leads to an increase in the relative concentration of primary versus secondary bile acids [9,16,17] and other microbe-associated metabolomic changes. These changes support favorable conditions for the spore germination and vegetative bacterial growth of *C. difficile* [18].

### 1.2. Current Therapies Do Not Address the Disrupted Microbiome

Currently approved treatment options for CDI include antibiotics that display activity against the vegetative form of *C. difficile* to treat the infection and monoclonal antibodies that bind to toxin-B for the prevention of rCDI. Vancomycin and fidaxomicin both have favorable minimal inhibitory concentrations against *C. difficile,* and achieve high stool concentrations resulting in symptom resolution within 3 to 5 days through the efficient killing of vegetative toxin-producing bacteria. However, these agents are not sporicidal and maintain or exacerbate the disrupted microbiome; this is particularly true for vancomycin [19]. Bezlotoxumab, a monoclonal antibody targeted against *C. difficile* toxin-B, is an adjunctive therapy to antibiotic treatment to reduce risk of rCDI in patients at high risk (e.g., the elderly and/or immunosuppressed, those with a high-risk ribotype, those with severe CDI, or those with a history of CDI in the last 6 months). However, neither antibiotics nor bezlotoxumab repair the disruption of the microbiome, which increases susceptibility to recurrent disease.

The recurrence of CDI following standard-of-care antibiotics highlights the need for a restorative microbiome therapeutic intervention, since patients with recurrence are at high risk for future episodes. Patients with primary CDI have a ~20–25% chance of recurrent infection. Recurrence rates in primary CDI after treatment with fidaxomicin were lower than that with vancomycin or metronidazole (15% [20]); however, the follow-up duration was short (i.e., 4 weeks). Recurrence rates are in the range of 20–36% among patients with first recurrence [21], and increase to ≥40% among those with ≥2 recurrences [8,15,22]. Moreover, the majority of CDI recurrences occur rapidly within a few weeks of antibiotic completion [7,15,23,24], highlighting the need for the rapid repair of the microbiome. To date, however, there are no microbiome restoration therapies approved by the U.S. Food and Drug Administration (FDA) for the treatment of recurrent CDI.

### 1.3. Fecal Microbiota Transplantation Provides Proof of Concept of the Importance of Microbiome Repair

Fecal microbiota transplant (FMT) studies suggest that microbiome restoration is key to a sustained clinical response. FMT involves the administration of minimally processed stool from a donor into the intestinal tract of a recipient [25,26,27]. Within days of FMT administration, microbial restoration is characterized by rapid gains of beneficial Firmicutes and reductions in proinflammatory Proteobacteria [26,28,29,30]. Sustained clinical responses are associated with the engraftment of Firmicutes bacteria [16,31,32,33,34].

However, the reported efficacy of FMT varies widely depending on the quality of the trial and delivery modality (i.e., enema vs. colonoscopy). A recent systematic review and meta-analysis reported considerably lower clinical cure rates in randomized controlled trials vs. open-label studies of FMT (weighted pooled rates: 67.7% vs. 82.7%, respectively); thus far, higher rates of efficacy have been generally reported with colonoscopy compared with enema delivery [35].

### 1.4. Safety Concerns Persist Regarding the Use of Fecal Microbiota Transplant

Current IDSA/SHEA guidelines recommend non-FDA-approved FMT only after 3 trials of antibiotics on the basis of “moderate quality of evidence”. The panel members expressed concerns about limitations in the evidence due to patient selection, prior treatments received, the duration of time from the last CDI episode, and risks of the transmission of emerging pathogens and/or microbes associated with acute and/or chronic infections [25,36].

The safety of FMT remains a concern, particularly given that it is unregulated. In addition, there is substantial heterogeneity in the methods used for donor selection, collection, and preparation of stool, and the route of administration [37]. Due to minimal processing, FMT can serve as a transmission vehicle for undetected infectious agents, highlighted in FDA safety alerts in 2019 after patients had received FMT contaminated with multidrug-resistant *E. coli* from a hospital-based program, and another FDA alert in 2020, when FMT from a stool bank had led to the transmission of Shiga toxin *E. coli* [38,39]. These events were associated with multiple hospitalizations and deaths [38,39,40].

FMT also has unforeseen risks due to its vulnerability to emerging infections [41,42]. The transmission of pathogens such as HIV, and hepatitis B and C via donor-derived biologic products occurred for years prior to clinical recognition and the development of sensitive screening assays to detect them. FMT from stool banks donated after 1 December 2019 was quarantined for months due to concerns relating to the potential transmission of SARS-CoV-2 [43], which has known receptors in the GI tract and can persist in feces for weeks after respiratory shedding has resolved [44,45]. However, the development and validation of an assay to detect SARS-CoV-2 in a stool matrix was not reported until several months had passed following the start of the COVID-19 pandemic [46]. Additionally, a new adenovirus strain linked to a cluster of severe pediatric hepatitis cases represents another pathogenic threat, given that the virus is primarily transmitted via the fecal–oral route [47]. Therefore, while FMT provides a proof of concept of the importance of microbiome restoration in reducing CDI recurrence. Using the full spectrum of microbes in stool brings risk and potential unintended consequences, highlighting the need for a more targeted and effective microbiome therapeutic that includes necessary risk mitigation measures for improved patient safety [42].

## 2. SER-109

SER-109, a potential first-in-class oral investigational microbiome therapeutic, was granted Breakthrough Therapy and Orphan Drug designations by the FDA for the treatment of recurrent CDI following standard-of-care antibiotics. SER-109 comprises live purified Firmicutes spores on the basis of their modulatory role in the life cycle of *C. difficile* and disease pathogenesis. Preclinical data demonstrated the efficacy of Firmicutes spores in reducing CDI recurrence [9], which led to the hypothesis that spore-forming bacteria may compete metabolically with *C. difficile* for essential nutrients and/or modulate bile acid profiles to re-establish colonization resistance.

### 2.1. Manufacturing and Characterization of SER-109

**Figure 2:**
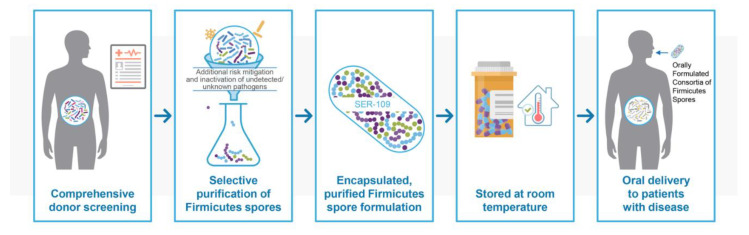
SER-109 Manufacturing processes mitigate risk.

A schematic of the manufacturing steps of SER-109 is shown in Figure 2. Manufacturing and quality systems for SER-109 are state-of-the-art, and were designed to deliver the microbial components that facilitate potency while mitigating the risk of pathogen transmission and significantly reducing the impurities associated with donor materials. The resilience of Firmicutes spores permits their enrichment through an ethanol-based purification process. Unlike most vegetative commensal organisms, spores are resistant to gastric acid, heat, and a range of chemical and physical changes, exhibiting exceptional stability during manufacturing and drug product storage, and allowing for oral delivery via capsules with a low pill burden.

Comprehensive donor screening and subsequent monitoring are essential first steps in producing the purified SER-109 product. Once a donor clears a thorough health history, physical examination, and full array of laboratory testing, stool is processed in a controlled bioprocessing environment through a proprietary, rigorous process designed to remove vegetative bacteria, fungi, parasites, and viruses via solvent inactivation and purification steps in compliance with current good manufacturing process regulations. The inactivation and clearance processes were further validated by challenge studies using model organisms of various pathogens. These studies confirmed the process’ capability to reduce potential pathogenic bacteria, fungi, parasites, and viruses to undetectable levels. Product release testing for SER-109 ensures conformance to microbiological purity standards [7,48].

This rigorous approach to drug manufacturing represents a philosophy of necessary redundancy to mitigate the risk to patients beyond donor screening alone, while enriching for a final product of highly purified Firmicutes spores in a convenient oral formulation with a low pill burden.

### 2.2. Efficacy and Safety

SER-109 efficacy and safety were evaluated in Phase 1b, 2b, and 3 trials. While the Phase 1b study provided proof-of-concept supporting the efficacy of a targeted, purified spore-based approach in patients with rCDI, the primary efficacy endpoint (reduction in CDI recurrence at Week 8 in SER-109 vs. placebo) in the Phase 2b trial was not achieved. An indepth investigation comparing the discordant results from the Phase 1b and 2 trials demonstrated that dosing in the Phase 2 study was suboptimal. In addition, the use of PCR diagnostic testing, which is less specific, may have resulted in the inclusion of colonized patients without active infection or may have led to a diagnosis of on-study recurrence in patients only colonized with *C. difficile*. Compared with subjects who had recurred, SER-109 species engraftment in the GI tract was significantly greater in subjects with a sustained clinical response (*p* < 0.05), affirming the association of engraftment with clinical outcome [7]. This analysis informed the design of the Phase 3 ECOSPOR-III trial, including a ~10-fold higher dose selection, and the requirement for diagnostic toxin testing at study entry and at suspected recurrence to ensure the enrollment of subjects with active CDI and confirm the accuracy of the endpoint.

ECOSPOR III was a double-blind placebo-controlled trial of 182 adults with three or more CDI episodes who were randomized to receive either SER-109 or placebo (4 oral capsules daily for three days) following standard-of-care (SOC) antibiotic treatment (vancomycin or fidaxomicin per investigator discretion). SER-109 was superior to the placebo in reducing CDI recurrence at Week 8, the primary endpoint (12% vs. 40%, respectively; relative risk (RR), 0.32 (95% CI, 0.18–0.58; *p* < 0.001 for RR < 1.0; *p* < 0.001 for RR < 0.833)) [8]. Of the SER-109 subjects, 88% met the alternative metric of a sustained clinical response compared with 60% of the placebo subjects [8].

Among subjects without a sustained clinical response, the time to recurrence was rapid, with the majority experiencing recurrence within the first month after dosing. There was also a significant treatment difference between the two arms in time to recurrence, showing an early clinical benefit of SER-109 compared with SOC antibiotics alone, which was maintained over 24 weeks [49].

The benefit of SER-109 over placebo was also consistently observed in analyses of subgroups of subjects with risk factors for recurrence. Regardless of age (<65 or ≥65 years), antibiotic received (vancomycin or fidaxomicin), or Charlson comorbidity score category, SER-109 following SOC antibiotics led to lower CDI recurrence rates compared with SOC antibiotics alone [50,51,52]. The efficacy of SER-109 was unaffected by the use of proton-pump inhibitors and H2-blockers, which are often discontinued in patients with CDI with subsequent risk of gastritis or other complications [53].

### 2.3. Safety Profile

Safety data from 231 patients treated with at least one dose of SER-109 across four clinical trials (SERES-001, SERES-004, SERES-005, and SERES-012) showed SER-109 to be well-tolerated. Most treatment-emergent adverse events (TEAEs) were mild-to-moderate in intensity and resolved without sequelae. The most common TEAEs in subjects who had received either SER-109 or the placebo were gastrointestinal in nature (e.g., flatulence, abdominal pain, abdominal distention, diarrhea, constipation). The most common TEAEs occurring at higher rates in SER-109 treated subjects compared to placebo-treated subjects were abdominal distension, constipation, diarrhea, and urinary tract infection. No urinary tract infections were related to SER-109, and available culture data showed expected uropathogens, unrelated to SER-109 dose species. In the placebo-controlled studies, the incidence of SAEs was similar between the SER-109 and placebo arms. No serious drug-related TEAEs were observed. Across these four clinical studies of 231 subjects who had received SER-109, there were 8 deaths (3.5%). This rate compares favorably to reported mortality rates in other treatment trials of patients with primary and recurrent CDI with rates up to 8.7% [54,55,56,57]. No deaths were considered to be related to SER-109. Fatalities were due to a range of causes, including those due to pre-existing conditions, with no detectable patterns. The observed safety profile of SER-109 might be anticipated since Firmicutes normally reside within the healthy microbiome.

### 2.4. Pharmacology of SER-109

Treatment with SER-109 leads to the engraftment of drug product species, driving compositional and functional changes in the microbiome thought to be critical to a sustained clinical response. These compositional and functional changes characterize the pharmacokinetics and pharmacodynamics of this unique microbiome therapeutic.

SER-109 Firmicutes spores germinate into metabolically active vegetative bacteria that colonize and replicate in the colon, a process referred to as engraftment, a measure of the pharmacokinetics of a microbiome drug. The engraftment of SER-109 dose species within the gastrointestinal tract leads to compositional changes in and the restructuring of the microbiome with reciprocal loss of proinflammatory Gram-negative Proteobacteria [58,59] as the abundance of Gram-positive Firmicutes increases. The engraftment of SER-109 dose species can be specifically tracked and quantified by examining the number of SER-109 dose species in post-treatment fecal samples, which were not present pretreatment using highly sensitive and specific genetic markers [7,8,48].

#### 2.4.1. Race to Microbiome Repair to Achieve a Sustained Clinical Response

In the vast majority of patients with recurrent CDI, the onset of symptom recurrence occurs quickly, in a matter of days or a few weeks after completion of antibiotic treatment. In ECOSPOR III, 65% of patients who experienced CDI recurrence during the study had onset of recurrence within one month of antibiotic discontinuation, underscoring the importance of a rapid and robust engraftment of SER-109 to achieve a sustained clinical response [49].

The rapid engraftment of SER-109 dose species (i.e., as early as one week following treatment initiation when patients are most at risk of recurrence) was observed in all clinical studies of SER-109 [7,8,48]. In placebo-treated subjects in ECOSPOR III, the number of Firmicutes species in fecal samples also increased over time, but this process was slow and insufficient to reduce the risk of recurrence during the critical window of vulnerability. In contrast, treatment with SER-109 led to faster engraftment, facilitating a sustained clinical response [8]

The magnitude of the engraftment of SER-109 dose species was also associated with clinical outcome [7,8]. In the Phase 2 trial, SER-109-treated subjects with a sustained clinical response had significantly more SER-109 dose-species engraftment than those who had recurred (*p* < 0.05) [7]. In ECOSPOR III, the magnitude of SER-109 engraftment in the SER-109 treated subjects was significantly greater than that in placebo-treated subjects as early as one week after dosing and was durable through Week 24 [8]. Thus, rapid engraftment is important to end the vicious cycle of recurrence, and the magnitude of engraftment may be key to durability of the clinical outcome.

#### 2.4.2. SER-109 Engraftment Drives Microbe-Associated Metabolite Changes, a Measure of the Pharmacodynamics of SER-109

**Figure 3:**
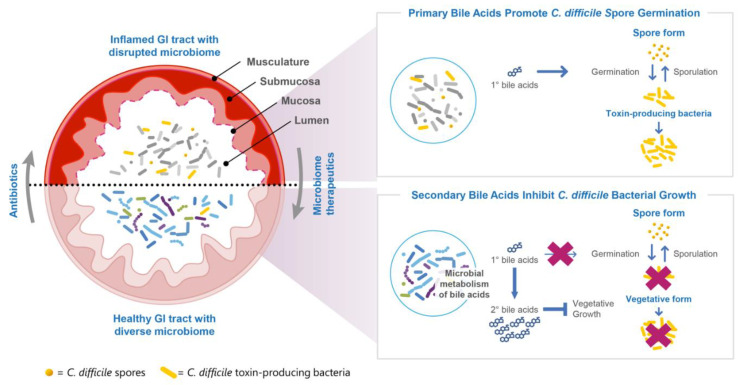
SER-109 engraftment and metabolomics.

The production of microbe-associated metabolites is relevant to multiple pathways hypothesized to govern CDI pathogenesis. Microbes modulate the relative concentrations of primary to secondary bile acids in the gut, which are believed to impact the life cycle of *C. difficile*. Studies suggest primary bile acids facilitate *C. difficile* spore germination into toxin-producing vegetative bacteria while secondary bile acids inhibit *C. difficile* spore germination and vegetative bacterial replication (Figure 3). These pathways are phylogenetically conserved and governed by members of the Firmicutes phylum.

In the Phase 2 trial, there was a significant positive correlation among the numbers of SER-109 dose species abundance of secondary bile acids, confirming the pharmacologic activity of SER-109 [7]. In ECOSPOR III, the concentrations of secondary bile acids rapidly increased from baseline in parallel with the number of engrafting SER-109 dose species as early as Week 1. Secondary bile acids were also significantly higher in the SER-109 arm compared with the placebo at all time points through Week 8 [8]. The low rate of CDI recurrences in the SER-109 arm observed in ECOSPOR III and these pharmacologic observations support the central role of Firmicutes-mediated conversion of primary to secondary bile acids in interrupting the two-phase life cycle of *C. difficile* and restoring colonization resistance [9] as one of the potential mechanisms of action supporting the efficacy of SER-109. Other possible mechanisms include the impact of free fatty acids, competition for nutrients, and reduced colonic inflammation.

### 2.5. Additional Potential Benefits of Restructuring the Gastrointestinal Microbiome

Patients with recurrent CDI often have a history of exposure to numerous courses of antibiotics, which puts them at risk of carrying antibiotic resistance genes (ARGs). The exposure to broad-spectrum antibiotics can lead to antimicrobial drug resistance, which can limit therapeutic options when infections arise. Drug-resistant bacteria can rapidly proliferate and become dominant in the gastrointestinal tract, particularly when the expansion of Gram-negative bacteria occurs in a vacuum of low microbial diversity. We hypothesized that restructuring the microbiome with SER-109 may have ancillary benefits on the prevalence and abundance of ARGs post treatment in stool samples of the study participants as compared to those who were randomized to placebo.

In fact, in this post hoc analysis of ECOSPOR III, ARGs were highly prevalent and abundant at the baseline among subjects in both arms, noted in multiple drug classes including fluoroquinolones, tetracyclines, and aminoglycosides [60].

However, SER-109 led to a restructuring of the microbiome, which was associated with changes in the prevalence and abundance of ARGs. SER-109 treatment was associated with a significant and rapid decline in ARGs as early as Week 1 compared to the placebo, with a sustained decline through Week 8 post-treatment [60]. A reduction in ARGs was associated with a marked decline in the relative abundance of the Proteobacteria phylum and an increase in Firmicutes. The analysis of the various bacterial families present revealed a marked decline in *Enterobactericeae*, which were positively associated with an abundance of ARGs. Non-spore-forming Firmicutes such as *Enterococcus* were also positively associated with ARGs. Conversely, the relative abundance of spore-forming Firmicutes found in SER-109 was negatively associated with ARG abundance, highlighting the potential advantage of this novel microbiome therapeutic over other “complete communities” that harbor both Proteobacteria and non-spore-forming Firmicutes [60].

The manufacturing process for SER-109 specifically retains spore-forming Firmicutes and excludes other organisms, including *Enterococcus* and *Enterobacteriaceae*. The effects of decreasing the reservoir of pathobionts that harbor clinically relevant ARGs may potentially minimize the horizontal gene transfer of ARGs to pathogens within host microbiomes, lower the shedding of ARGs into the environment, and reduce the risk of antibiotic drug-resistant bacterial infections. Further research into these potential benefits is warranted in light of the limited pipeline of drugs for antibiotic-resistant infections.

## 3. Conclusions

CDI is the result of a two-hit process involving microbiome disruption and exposure to *C. difficile* spores. Antibiotic treatment is necessary but often insufficient, leading to a cycle of recurrent infection due to persistent microbiome dysfunction.

SER-109 is a potential first-in-class oral investigational microbiome therapeutic comprised of purified Firmicutes spores designed to repair the disrupted microbiome after treatment with standard-of-care antibiotics. Clinical data from a rigorous well-designed Phase 3 clinical trial, ECOSPOR III, demonstrate the superiority of SER-109 compared with placebo in reducing CDI recurrence in adults with a history of rCDI with an observed favorable safety profile. Additionally, the manufacturing process of SER-109 removes unwanted microbes, thereby mitigating the risk of pathogen transmission beyond donor screening alone.

A two-pronged treatment approach including antibiotics to kill *C. difficile* bacteria, followed by SER-109 to address microbiome disruption, may represent a potential paradigm shift in the clinical management of patients with recurrent CDI.

## Data Availability

Not applicable.
